# Improving the electrical properties of graphene layers by chemical doping

**DOI:** 10.1088/1468-6996/15/5/055004

**Published:** 2014-09-08

**Authors:** Muhammad Farooq Khan, Muhammad Zahir Iqbal, Muhammad Waqas Iqbal, Jonghwa Eom

**Affiliations:** Department of Physics and Graphene Research Institute, Sejong University, Seoul 143-747, Korea

**Keywords:** graphene, potassium nitrate, chemical doping, electrical properties, Raman spectroscopy

## Abstract

Although the electronic properties of graphene layers can be modulated by various doping techniques, most of doping methods cost degradation of structural uniqueness or electrical mobility. It is matter of huge concern to develop a technique to improve the electrical properties of graphene while sustaining its superior properties. Here, we report the modification of electrical properties of single- bi- and trilayer graphene by chemical reaction with potassium nitrate (KNO_3_) solution. Raman spectroscopy and electrical transport measurements showed the n-doping effect of graphene by KNO_3_. The effect was most dominant in single layer graphene, and the mobility of single layer graphene was improved by the factor of more than 3. The chemical doping by using KNO_3_ provides a facile approach to improve the electrical properties of graphene layers sustaining their unique characteristics.

## 1. Introduction

Graphene, an exemplary two dimensional carbon system, is one of the most promising materials for future electronic applications [1–3]. The fascinating properties of graphene are utilized in a variety of fields in nanoelectronics [1, 4], but a feature that charge carriers are constrained to a plane of atomic thickness makes the graphene devices more responsive to surrounding environment like substrate [5, 6], dielectric medium in contact with graphene [7–10], and surface charges [11–13]. A control of surrounding environment becomes demanding technology to manipulate graphene properties. Doping is an especially striking approach to tailor and control the properties of graphene. The modulation of Fermi level of graphene through doping such as metal doping [14], electrostatic back-gate doping through substrate [15–17], chemical doping [18, 19] and electrochemical doping [20, 21] are simple ways to control transport, structural and optical properties.

Another important theme in current graphene-based research is to explore the doping effect of graphene with different number of layers, where the band structures are distinct [22, 23]. The study of multilayer graphene is also meaningful for transparent conducting electrodes as stacking of graphene layers reduces the sheet resistance [3, 24–27].

Potassium (K) doping plays an important role in adjusting the electronic properties of carbon materials. The potassium atoms donate their valence electrons to the graphene surface layer, making the n-type doping effect. There were a number of reports on the doping effect of graphene by deposition of potassium atoms in vacuum [28–30]. There were also reports on the bulk potassium graphite intercalation compounds made by vapor transport methods [31, 32]. Although various methods have been employed to make potassium doping on graphene, it has yet to be achieved by chemical doping method. Chemical doping is easy and even applicable to a graphene device without disturbing the structural shape. In this paper we made potassium doping of graphene by using potassium nitrate (KNO_3_) solution which was dissolved in deionized water. Potassium ions in aqueous solution adsorbed on the top surface of graphene inducing the n-type doping effect.

The chemical doping of graphene with KNO_3_ solution in this paper provides unique advantages because it enhances the mobility of graphene layers without inducing defects in graphene as we confirmed by Raman spectroscopy. However, the adsorbed molecules form covalent bonding with graphene surface in some other chemical doping methods. The covalent bonding between chemical species and carbon atoms changes basic electronic structure of graphene and causes substantial reduction of the carrier mobility [14, 33–35]. Non-covalent association of chemical species with graphene has been known to modify the electronic properties without introducing much deterioration, but it still reduces the charge carrier mobility and induces defects in graphene [19, 36–41].

Here we report modification of electrical properties of mechanically exfoliated single layer graphene (SLG), bilayer graphene (BLG) and trilayer graphene (TLG) field-effect transistors (FETs) by chemical reaction with KNO_3_ solution. The electrical transport measurements and Raman spectroscopy techniques revealed that chemical reaction with KNO_3_ induced n-doping of graphene and reduced the impurity scattering to enhance the performance in SLG, BLG and TLG devices. The n-doping effect was enhanced with increasing the reaction time. This kind of chemical doping provides a facile approach to tune the electrical properties of graphene layers.

## 2. Experimental section

### 2.1. Preparation of graphenes

**Figure 1.**
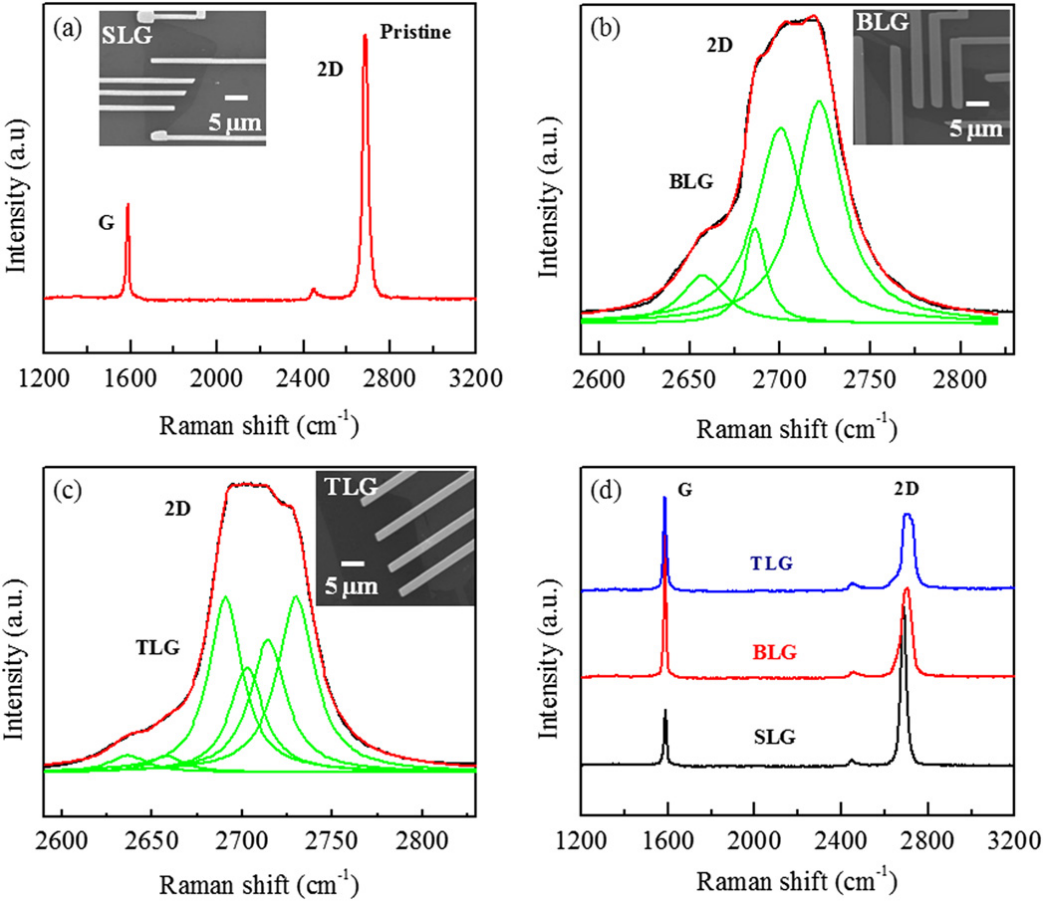
(a) Raman Spectrum of SLG. Lorentzian curve fitting of (b) BLG and (c) TLG. (d) Comparative Raman spectra of pristine SLG, BLG and TLG. Insets in **a**, **b** and **c** show SEM images of SLG, BLG and TLG devices.

The SLG, BLG and TLG were obtained by mechanical exfoliation of natural graphite flakes by using the Scotch tape and then transferred on Si/SiO_2_ wafer [1]. These graphene layers were identified by optical microscopy and Raman spectroscopy. The big patterns were made by photolithography and then Cr/Au (6/30 nm) were deposited by thermal evaporation technique for all graphene layers on Si/SiO_2_ substrate. For complete device fabrication and electrical measurements the fine electrodes were made by e-beam lithography and then Cr/Au (12/60 nm) were also deposited by thermal evaporation technique. The scanning electron microscopy (SEM) images of final graphene devices are shown in the insets of figure 1.

### 2.2. Potassium nitrate doping and characterization

The modification of graphene properties by KNO_3_ was investigated by electrical transport measurements and Raman spectroscopy. Measurements were performed on the same devices before and after different reaction time of KNO_3_ treatment for SLG, BLG and TLG samples. Potassium nitrate is a chemical compound and an ionic salt of potassium ions K^+^ and nitrate ions NO_3_^−^. It was dissolved in deionized water to make the concentration of 0.1 M solution. The Raman spectra were taken at room temperature with a Renishaw microspectrometer. A 514 nm laser was used for excitation, and its power was kept at 1.0 mW to minimize the laser-induced heating effect. The area of laser spot is around 1 *μ*m^2^ and the spectral resolution is better than 1 cm^−1^. The SLG, BLG and TLG samples were dipped in the KNO_3_ solution for a certain reaction time and then dried with nitrogen gas and heated at 100 °C on hot plate for 2 min. Furthermore, we put these samples in vacuum for 2 h to completely dry. The Dirac points of graphene layers were examined in vacuum by gate voltage dependent resistivity measurement using 4-probe configuration with standard lock-in amplifier.

## 3. Results and discussion

Raman spectroscopy is a standard nondestructive and fast tool to characterize and identify the number of graphene layers [42, 43]. Figure 1(a) shows the Raman spectra of pristine SLG. The characteristic G and 2D peaks appear around 1589 and 2687 cm^−1^, respectively. The intensity ratio of 2D and G peaks is found to be 2.6 which confirms the signature of SLG. In figure 1(b) a broad 2D peak is fitted with four Lorentzian curves, confirming the BLG structure. Figure 1(c) shows a six-Lorentzian curve fitting of the broad 2D band of TLG. Figure 1(d) shows the comparative Raman spectra of pristine SLG, BLG and TLG. The characteristic G and 2D peaks for BLG and TLG are observed around 1587 and 2705 cm^−1^ and 1586 and 2710 cm^−1^, respectively. The absence of D peak in SLG, BLG, and TLG is a signature of high quality of graphene layers.

**Figure 2.**
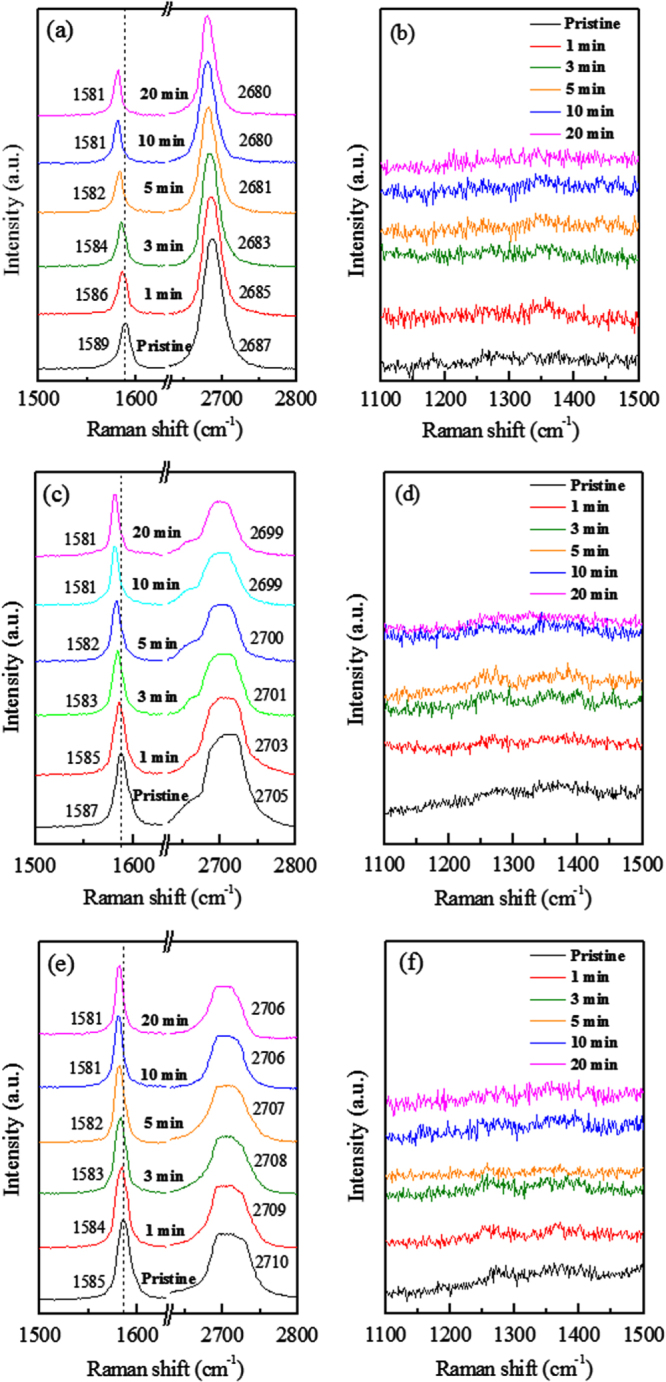
Raman spectra of (a) pristine and KNO_3_-doped SLG, (c) pristine and KNO_3_-doped BLG and (e) Pristine and KNO_3_-doped TLG. (b), (d) and (f) depict the absence of D peak of pristine and KNO_3_-doped SLG, BLG and TLG, respectively. The colors coding of panels **a**, **c** and **e** are same as panel **b, d** and **f**, respectively.

Figure 2 shows the Raman spectra of SLG, BLG and TLG before and after treatment of KNO_3_ for different reaction times (1, 3, 5, 10, and 20 min). It is already reported that the shift of the G and 2D peaks positions toward lower wavenumbers is attributed to n-type doping [34, 40, 44, 45], and the shift of the G and 2D peaks positions toward higher wavenumbers is attributed to p-type doping in graphene layers [46–48]. Figure 2(a) shows the Raman spectra of pristine and KNO_3_-doped SLG. A red shift in the G and 2D peak positions is observed after KNO_3_ treatment, the shift increases with increasing the reaction time.

Figure 2(c) shows the Raman spectra of BLG treated for different periods of time. It reveals a shift in the G peak position toward lower wavenumbers compared with pristine BLG. The red shift in G peak position increases with increasing the treatment time. The shifting of G peak position toward lower wavenumber also demonstrates the n-doping of BLG. Figure 2(e) shows the Raman spectra of TLG treated for different periods. The red shift is also observed in G peak position in TLG, and increases with increasing the reaction time. The shift of the G peak position toward lower wavenumbers with increasing the reactions time is smaller as compared to SLG and BLG. The 2D peak positions of SLG, BLG and TLG of Raman spectra before and after KNO_3_-doping are also shown in figures 2(a), (c) and (e), respectively. Figures 2(b), (d) and (f) show the Raman spectra of SLG, BLG and TLG, respectively to see the clear absence of D peak.

**Figure 3.**
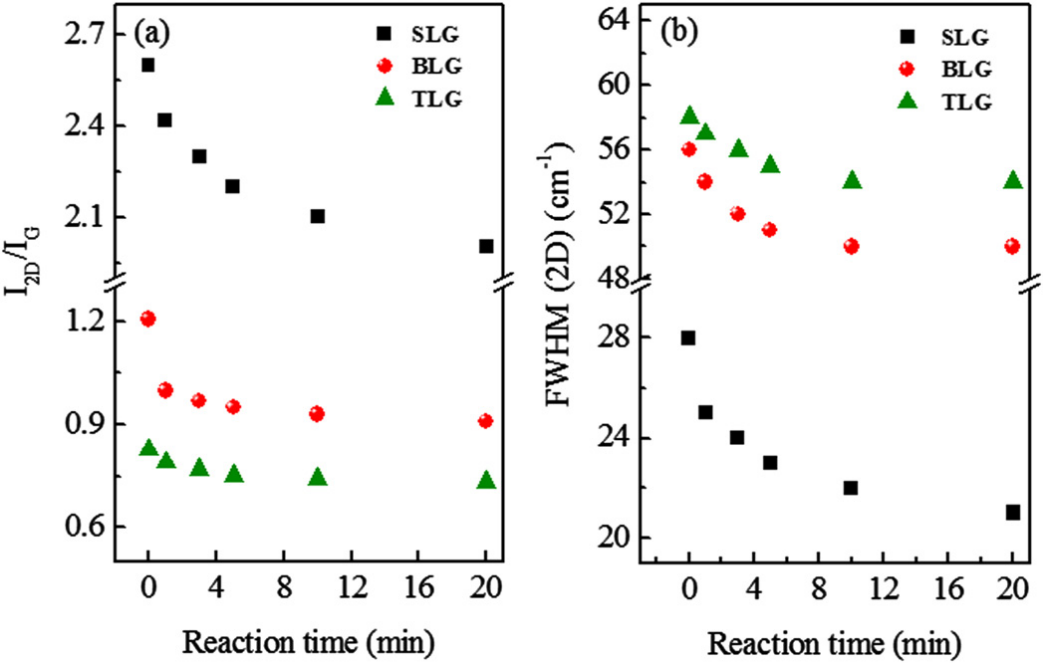
(a) Ratios of intensities of SLG, BLG and TLG as function of reactions time. (b) Full width at half maxima (FWHM) of 2D band of Raman Spectra of SLG, BLG and TLG as a function of reaction time.

Figure 3(a) shows the intensity ratio of 2D and G peak (I_2D_/I_G_) before and after KNO_3_ treatment for SLG, BLG and TLG for different treatment time. The I_2D_/I_G_ decreases with increasing of the reaction time for SLG, BLG and TLG. This reduction indicates the doping of graphene layers with KNO_3_ [20, 23, 49]. Figure 3(b) shows the full width half maximum (FWHM) of 2D band of Raman spectra for SLG, BLG and TLG before and after KNO_3_ treatment. The FWHM decreases with increasing of the treatment time by KNO_3_, which indicates that impurity scatterings are suppressed [50]. In addition, we did not observe any D peak in our results. So the absence of D peak indicates that KNO_3_ did not change the band structure of graphene layers and made non-covalent bonding with carbon atoms [35].

**Figure 4.**
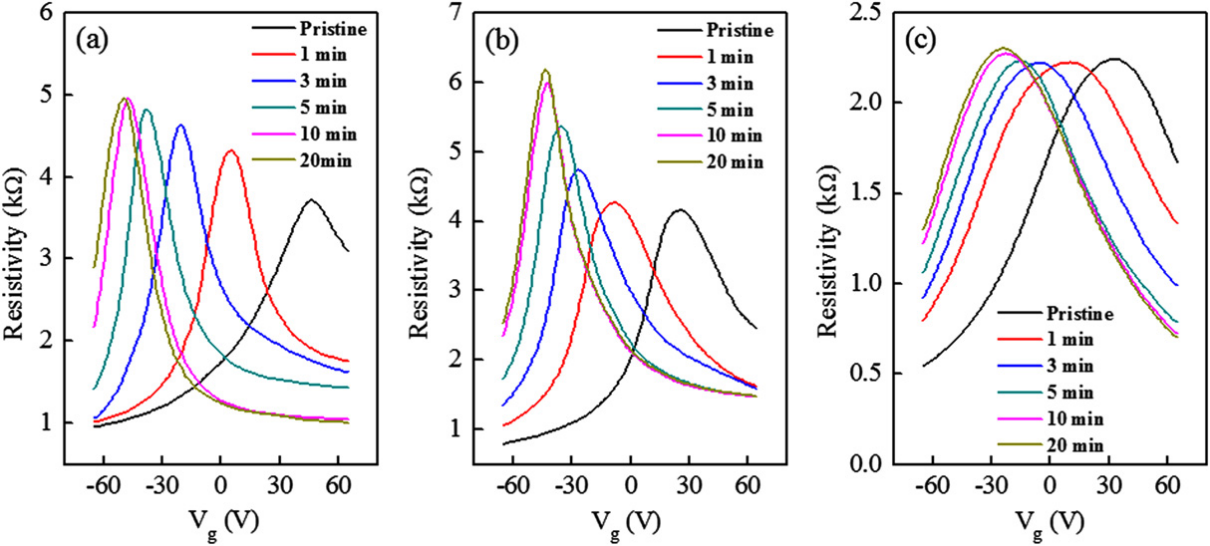
Resistivity as a function of back gate voltage (V_g_) for (a) SLG before and after KNO_3_ treatment for different reaction time, (b) BLG before and after KNO_3_ treatment for different reaction time and (c) TLG before and after KNO_3_ treatment for different reaction time.

The n-doping effect of exfoliated SLG, BLG, and TLG is also confirmed by electrical transport measurements. The resistivity as a function of gate voltage (V_g_) before and after KNO_3_ treatment of SLG, BLG and TLG is shown in figure 4. Figure 4(a) shows the shift of Dirac point (V_Dirac_) of SLG towards negative gate voltage as we increase the reaction time. The Dirac point of pristine SLG was observed at +47 V and then shifted towards +4 V after 1 min treatment time. The shift increased gradually with increasing treatment time and was saturated at −49 V after 20 min treatment. Figures 4(b) and (c) show the Dirac point shift of BLG and TLG, respectively, for different reaction times. The Dirac point of pristine BLG appeared at +25 V and shifted to −8V after 1 min treatment. The shift increased progressively with increasing doping time and was saturated at −43 V after 20 min treatment. Finally, the Dirac point of pristine TLG was around +33 V. After 1 min reaction the Dirac point of TLG reached at +8 V and was saturated at −23 V after 20 min treatment. The shifting of Dirac point toward negative gate voltage is a clear indication of n-doping of the exfoliated SLG, BLG and TLG. The total change in Dirac point of SLG, BLG, and TLG after 20 min treatment is 96 V, 68 V, and 56 V, respectively. The shift of Dirac point is relatively large in SLG as compared to BLG and TLG. Obviously, the doping effect is dominant on the top surface of graphene layer as compared to the underneath layers. However, the doping effect is still significant for BLG and TLG.

If graphene is perfect and contains no dislocations or impurities, it should show an infinite resistance at the charge neutrality point. However, a real graphene always shows a finite resistance at the charge neutrality point, where electron-hole puddles exist due to charge impurities. The charge impurities are believed to generate inhomogeneous potential fluctuations that create electron-hole puddles in graphene [51, 52]. The increase of the maximum resistance at the charge neutrality point after the chemical doping by KNO_3_ solution is related with the reduction of charge impurities, which yields to improvement of electrical mobility in graphene. Since only the top surface of graphene device is in contact with KNO_3_ solution, the reduction of charge impurities is less effective at the graphene layers which exist underneath the top layer. Therefore, the change of resistance decreases as number of graphene layers increases. We note that after KNO_3_ treatment the residual resistance of graphene away from the charge neutrality point is smallest for SLG as seen in figure 4(a).

**Figure 5.**
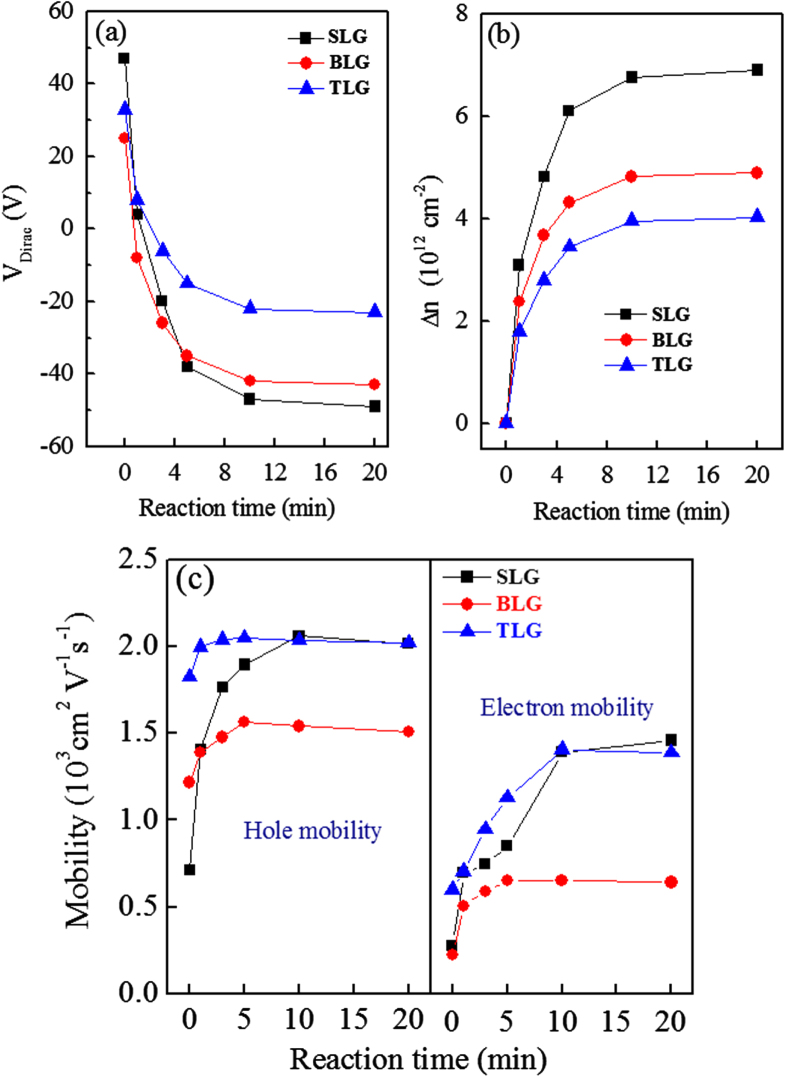
(a) Shift in Dirac point positions of SLG, BLG and TLG devices as a function of reaction time. (b) Change in charge density (*Δ*n) as a function of KNO_3_ reaction time for SLG, BLG, and TLG. (c) The electron and hole mobility as a function of KNO_3_ reaction time for SLG, BLG, and TLG.

Figure 5(a) shows the comparative trend of shift in Dirac points of SLG, BLG and TLG as function of reaction time. The saturation of Dirac point shift depends on the adsorption limit of potassium ions on graphene surface. When the graphene surface is completely adsorbed after a certain doping time then the shift of Dirac point becomes saturated. The shift of Dirac point is smallest in TLG because the number of graphene layers which are not in direct contact with KNO_3_ solution is largest in TLG.

Figure 5(b) shows the change in charge carrier density (*Δ*n) of SLG, BLG, and TLG as a function of KNO_3_ reaction time. Figure 5(b) evidently shows that charge carrier density of SLG, BLG, and TLG changes significantly after time by KNO_3_ treatment. The changes in charge carrier density (*Δ*n) of graphene layers were estimated by using the relation *Δ*n = C_g_(V_g_ − V_Dirac_)/e, where C_g_ is the gate capacitance ∼115 aF *μ*m^−2^ for our Si/SiO_2_ substrate, V_Dirac_ is the Dirac point of SLG, BLG, and TLG, V_g_ is the back gate voltage and ‘e’ is the electronic charge. The change in charge carrier density of graphene layers is related with change in Fermi level of graphene layers by KNO_3_-doping.

Figure 5(c) shows mobilities of electrons and holes in SLG, BLG, and TLG as a function of the KNO_3_ reaction time. The field effect mobilities of the different graphene layers were obtained using relation *μ* = (1/C_g_) (∂*σ*/∂V_g_), where *σ* = 1/*ρ* is the conductivity of samples. The mobility of pristine and doped graphene was calculated on the basis of slope fitted to the linear region of their respective conductivity data. The charge carrier mobility of SLG, BLG and TLG increases with increasing the treatment time. The electron mobility of SLG, BLG and TLG was improved by 424%, 178% and 130% respectively after 20 min reaction with KNO_3_ solution. Similarly the hole mobility of SLG, BLG and TLG was enhanced by 183%, 23% and 11% respectively after 20 min reaction with KNO_3_ solution. The mobility improvement was most dominant for SLG because the entire graphene layer is directly exposed to KNO_3_ solution. This is a surprising result, since the doping treatments of graphene mostly have shown reduction of mobility [40, 45, 53, 54]. The mobility improvement may be due to the ionic screening of charged-impurities scattering [7]. Our results demonstrate that the performance of graphene devices is enhanced by chemical doping of potassium compound.

## 4. Conclusions

We have investigated the electrical properties of mechanically exfoliated SLG, BLG and TLG by reaction with KNO_3_ solution. The electrical transport measurements and Raman spectroscopy confirmed that KNO_3_ imposes the n-doping for all graphene layers. The shift of G and 2D band positions and intensity ratios of I_2D_/I_G_ for SLG, BLG and TLG are analyzed for different reaction times. The magnitude of I_2D_/I_G_ for SLG, BLG, and TLG decreases as the doping effect increases. For all graphene layers the D peak intensity does not increase after the reaction with KNO_3_ solution. So this indicates that KNO_3_ does not change the band structure of graphene layers. The shift of Dirac points toward negative gate voltage also confirmed the n-doping by KNO_3_ treatment. The mobilities have been found to be gradually improved with reaction time. These results indicate that chemical modification is a useful approach to tailor the electrical properties of graphene layers while enhancing the mobility of graphene FET devices. Chemical doping using KNO_3_ can play an important role in modulating the electronic properties of graphene layers for future graphene-based transparent electronics.
